# Analysis of fetal heart rate fluctuations in women diagnosed with preeclampsia during the latent phase of labor

**DOI:** 10.3389/fphys.2024.1340441

**Published:** 2024-05-23

**Authors:** Nancy B. Lucero-Orozco, José Javier Reyes-Lagos, María del Rocío Ortíz-Pedroza, Ana Karen Talavera-Peña, Eric Alonso Abarca-Castro, Hugo Mendieta-Zerón, Adriana Cristina Pliego-Carrillo, Jorge Rodríguez-Arce, Luis Adrián Zúñiga-Avilés, Laura Mercedes Santiago-Fuentes, Claudia Ivette Ledesma-Ramírez, Miguel Ángel Peña-Castillo

**Affiliations:** ^1^ División de Ciencias Básicas e Ingeniería, Universidad Autónoma Metropolitana-Iztapalapa (UAM-I), Ciudad de México, Mexico; ^2^ Facultad de Medicina, Universidad Autónoma del Estado de México (UAEMéx), Toluca, Mexico; ^3^ Departamento de Ciencias de la Salud, Universidad Autónoma Metropolitana-Lerma (UAM-L), Lerma de Villada, Mexico; ^4^ Facultad de Ingeniería, Universidad Autónoma del Estado de México (UAEMéx), Toluca, Mexico; ^5^ Departamento de Ciencias de la Salud, Universidad Autónoma Metropolitana-Iztapalapa (UAM-I), Iztapalapa, Mexico

**Keywords:** preeclampsia, labor, fetal heart rate variability, autonomic nervous system, complexity

## Abstract

**Introduction:**

Fetal heart rate variability (fHRV) is a tool used to investigate the functioning of the fetal autonomic nervous system. Despite the significance of preeclampsia, fHRV during the latent phase of labor has not been extensively studied. This study aimed to evaluate fetal cardiac autonomic activity by using linear and nonlinear indices of fHRV analysis in women diagnosed with preeclampsia without hypertensive treatment during gestation, compared to normotensive women during the latent phase of labor.

**Methods:**

A cross-sectional and exploratory study was conducted among pregnant women in the latent phase of labor, forming three study groups: normotensive or control (C, 38.8 ± 1.3 weeks of pregnancy, n = 22), preeclampsia with moderate features (P, 37.6 ± 1.4 weeks of pregnancy n = 10), and preeclampsia with severe features (SP, 36.9 ± 1.2 weeks of pregnancy, n = 12). None of the participants received anti-hypertensive treatment during their pregnancy. Linear and nonlinear features of beat-to-beat fHRV, including temporal, frequency, symbolic dynamics, and entropy measures, were analyzed to compare normotensive and preeclamptic groups.

**Results:**

Significantly lower values of multiscale entropy (MSE) and short-term complexity index (C_i_) were observed in the preeclamptic groups compared to the C group (*p* < 0.05). Additionally, higher values of SDNN (standard deviation of R-R intervals) and higher values of low-frequency power (LF) were found in the P group compared to the C group.

**Conclusion:**

Our findings indicate that changes in the complexity of fetal heart rate fluctuations may indicate possible disruptions in the autonomic nervous system of fetuses in groups affected by undiagnosed preeclampsia during pregnancy. Reduced complexity and shifts in fetal autonomic cardiac activity could be associated with preeclampsia’s pathophysiological mechanisms during the latent phase of labor.

## 1 Introduction

Fetal monitoring plays a pivotal role in the detection of maternal and fetal disorders (Varela and Peiro, 2021). Additionally, the importance of prenatal care throughout pregnancy, labor, and after childbirth has been emphasized by the World Health Organization (WHO) as conveyed by Tunçalp et al. (2015). It is worth noting that the World Health Organization (2023) report underscores that approximately 95% of maternal deaths that year occurred in low- and middle-income countries, emphasizing the potential for prevention through the provision of quality prenatal care. This gap in care can result in untreated maternal conditions, such as preeclampsia (PE). In cases of PE, diagnosis can be established with the onset of hypertension (defined as SBP ≥140 mmHg and/or DBP ≥90 mmHg) after the 20th week of gestation, either with significant proteinuria (>300 mg/day or a protein/creatinine ratio >0.3 mg/mg) or, in the absence of proteinuria, through the new onset of hypertension and signs of organ dysfunction such as thrombocytopenia, hepatic alterations, renal insufficiency, pulmonary edema, or neurological symptoms (Rodríguez Benítez, 2020). This maternal condition impacts fetal development within the uterus, often leading to fetal distress (Salas Ramírez et al., 2020).

During a healthy pregnancy, the fetal autonomic nervous system can mount an appropriate response to the demands imposed by the stress of labor. During the latent phase of labor, brief episodes of hypoxia prompt the fetus to undergo cardiovascular, metabolic, and respiratory adjustments, facilitating its readiness to survive outside the womb (Langer et al., 2017). A quantitative methodology for evaluating fetal wellbeing involves the analysis of fetal autonomic activity through fetal heart rate variability (fHRV). The fHRV is intricately linked to the development and operation of the fetal autonomic nervous system (SNA), elucidating fluctuations mediated by sympathetic and parasympathetic activity (Ponsiglione et al., 2021). Montalvo-Jaramillo et al. (2020) found that cardiac variability decreases during the active phase of labor and sympathetic activity increases, as determined by both linear and nonlinear indices of fHRV. These findings suggest that the fetus experiences significant autonomic changes during labor.

Quantifying the complexity of cardiovascular regulation through heart rate variability analysis remains challenging. In this context, methodologies embracing the multiscale nature of signals, such as Multiscale Entropy (MSE), have gained prominence. These approaches underscore the importance of examining control and pathological groups across various temporal scales, extending beyond the shortest scale (i.e., scale factor τ = 1). Furthermore, it is crucial to consider specific entropy values and evaluate how they change across different time scales, as the relationship between entropy and scale factor varies with the underlying pathological conditions (Silva et al., 2016).

Preclinical studies on ovine fetuses exposed to hypoxic-ischemic conditions demonstrated significant changes in fHRV metrics following umbilical cord occlusions that simulate human labor. This included elevated vagal-related indices such as High Frequency (HF) and Root Mean Square of Successive Differences (RMSSD), as reported by Frasch et al. (2016) and Kasai et al. (2019). This paradoxical increase in vagal activity is linked to systemic and brain inflammatory responses, including changes in HMGB1 distribution within microglia, suggesting an activation of the cholinergic anti-inflammatory pathway. In fetuses of mothers diagnosed with PE, particularly those who receive anti-hypertensive treatment during the third trimester of pregnancy, sympathetic overstimulation has been observed, potentially indicating fetal distress (Lakhno, 2017). This phenomenon highlights the significant impact of PE on the physiological processes of pregnancy. Despite the prudent use of antihypertensive medications, achieving autonomic balance in cases of PE remains challenging. Moreover, nonlinear indices have been reported to complement the analysis of fluctuations detected in the linear analysis of fHRV (Ponsiglione et al., 2021; Reyes-Lagos and Abarca-Castro, 2021).

Significant knowledge gaps persist, especially in developing countries where comprehensive prenatal care may be lacking. In such regions, pregnant women often receive their diagnosis of PE only when they reach the critical stage of labor (Organización Panamericana de la Salud and Organización Mundial de la Salud, 2018), considering that many of the signs and symptoms are not noticed until the condition manifests in its acute stage (Armaly et al., 2018). The absence of early detection and the limited access to prenatal monitoring in these resource-constrained settings pose serious concerns. This knowledge void leaves us with questions about how fetuses, in the absence of appropriate prenatal care, cope with the challenges posed by PE during labor. Furthermore, it is imperative to understand the impact of this delayed diagnosis on fetal wellbeing, particularly during the latent phase of labor when the fetus is exposed to brief episodes of hypoxia. Improving our understanding of these dynamics is critical for enhancing mothers’ and infants’ care and outcomes in regions where prenatal monitoring is insufficient or inaccessible.

This study aims to investigate changes in fetal autonomic cardiac activity and fetal heart rate complexity in parturient women with preeclampsia, both with and without severe features, who were not diagnosed until the onset of labor, and who did not receive antihypertensive treatment during pregnancy. Given that fetuses exposed to any severity level of PE and undergoing antihypertensive treatment have shown a distinct autonomic response compared to healthy fetuses—characterized by increased sympathetic activity and decreased parasympathetic activity—it is hypothesized that fetuses not diagnosed with PE until the onset of labor will exhibit a different autonomic behavior compared to those of normotensive pregnant women. Reduced complexity in heart rate variability may be evidence of this difference. The reduced complexity and potential cardiac autonomic changes may be attributed to their limited ability to adapt to periods of fetal hypoxia generated during the stage of labor.

## 2 Materials and methods

### 2.1 Participants

We conducted our research at the Emergency and Obstetrical Surgery Department of the Maternal-Perinatal Hospital “Mónica Pretelini Sáenz” in Toluca de Lerdo, Mexico State, Mexico, from April 2022 to August 2023. During this period, we selected pregnant Mexican women aged between 17 and 41 years, specifically at the third trimester of pregnancy. Before we collected any physiological data, we ensured that all participants were well-informed about the study’s objectives, and we obtained informed consent from those who willingly volunteered to participate. Our study protocol received approval from the institution’s Research Ethics Committee, with registration number 2021-03-719, and we meticulously adhered to all relevant institutional and general ethical guidelines during its implementation.

The participants were divided into three groups: preeclampsia without severe features or mild preeclampsia (P, n = 10), preeclampsia with severe features (SP, n = 12), and normotensive control women (C, n = 22). The medical doctors at the Maternal-Perinatal Hospital were responsible for determining the seriousness of preeclampsia. They followed the specific criteria to diagnose preeclampsia, categorizing it as mild-moderate preeclampsia when systolic blood pressure (SBP) ranged between 140 and 149 mmHg and diastolic blood pressure (DBP) was between 90 and 99 mmHg, in conjunction with the presence of proteinuria (a protein/creatinine ratio of 0.28–0.29). Severe preeclampsia was defined when SBP exceeded 150 mmHg, DBP was higher than 100 mmHg, and a protein/creatinine ratio was greater than 0.3, following the criteria established by the ACOG criteria (The American College of Obstetricians and Gynecologists, 2022).

Our study included pregnant women during the latent phase of labor, a period that spans from the onset of labor to the commencement of the active phase. Exclusion criteria for all groups included the presence of chronic or gestational hypertension without an accompanying protein/creatinine ratio indicative of proteinuria, diabetes mellitus, autoimmune, renal, or cardiovascular diseases, twin pregnancy, and the administration of epidural blockade during labor. It is relevant to mention that upon arriving at the Emergency Department with latent labor symptoms, the medical and nursing team noted high blood pressure in the patients. It led to immediate lab tests for proteinuria, a crucial sign of preeclampsia. These women had not attended any prenatal care appointments, leaving their preeclampsia undiagnosed until now. Detecting proteinuria to confirm preeclampsia is standard procedure for handling such cases at this hospital. While waiting for lab results, we recorded physiological signals per our study’s inclusion criteria. Once proteinuria was confirmed, indicating preeclampsia, we included these patients in our research and proceeded with detailed data collection. For normotensive participants, data on urinary creatinine and microproteins are not available, as these tests were only conducted when participants exhibited high blood pressure values.

Participants followed a controlled diet during the assessment, allowing water consumption. Additionally, due to their pregnancy status and adherence to hospital guidelines, they were advised to refrain from consuming caffeine or any substances that might potentially affect the fetus.

### 2.2 Data acquisition and preprocessing

**FIGURE 1:**
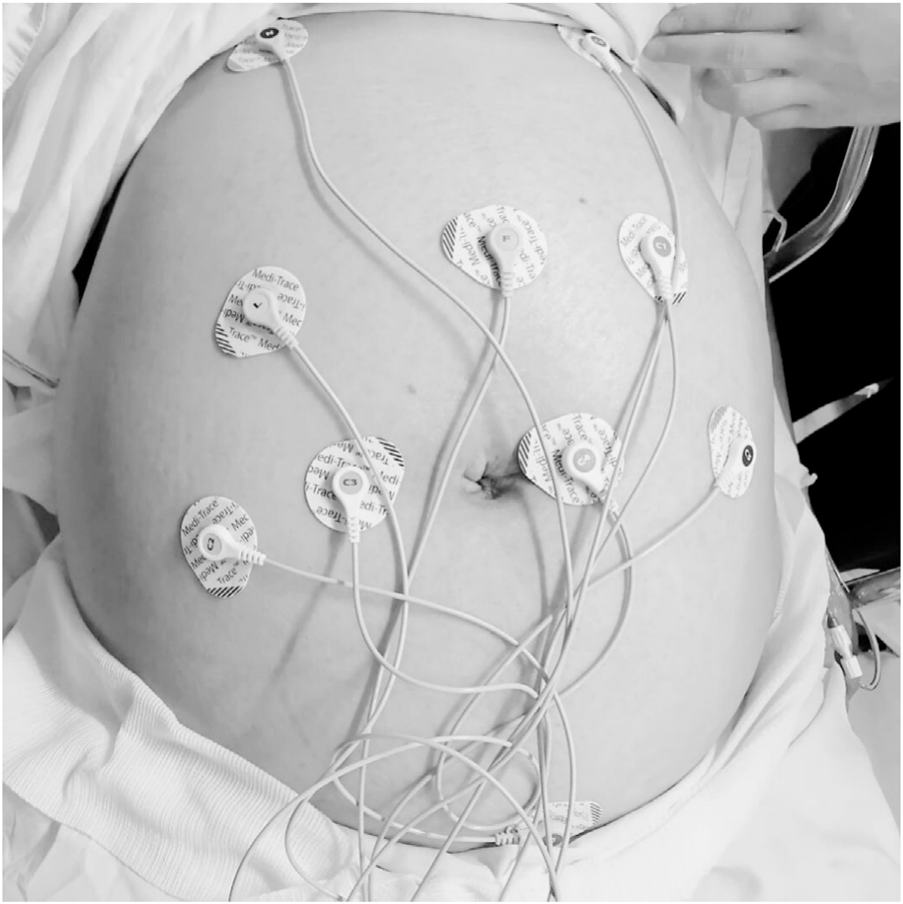
Experimental setup for data acquisition from transabdominal recordings using the BabyCard equipment in parturient women.

**FIGURE 2:**
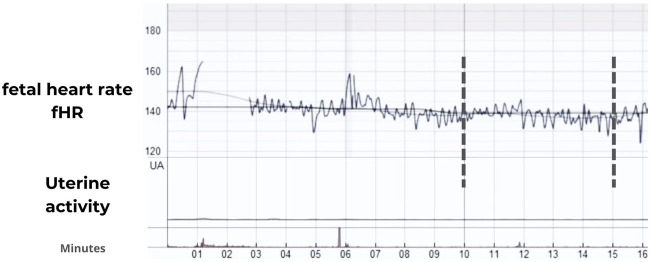
Representative fetal heart rate (fHR) traces in bpm during the latent stage of the labor in the severe preeclampsia (SP) group using the Cardiolab software (Scientific and Research Center “KhAI Medica,” Ukraine). Vertical marks indicate an example of 5 min of continuous recording.

The noninvasive fetal electrocardiogram was acquired for 30 min from the maternal abdominal wall using the BabyCard equipment (Scientific and Research Center “KhAI Medica,” Ukraine) (Viunytskyi and Shulgin, 2017a), with a sampling rate of 1,000 Hz with a 16-bit resolution and a range of ±8 mV. The recordings were conducted during latent labor in a semi-Fowler position. Ten electrodes were placed on the maternal abdominal wall for transabdominal recording while at rest (Figure 1). This setup utilized a monopolar lead system comprising seven active abdominal electrodes, one chest electrode for clear maternal electrocardiogram (MECG) recording, one reference (common) electrode, and one ground electrode. Subsequently, the fetal R-R interval was calculated using the Cardiolab software (Scientific and Research Center “KhAI Medica,” Ukraine). This software analyzes fetal electrocardiogram (fECG) data, which is obtained from the abdominal wall. It employs real-time analysis through algorithms across seven channels. The process involves several steps: detection of MECG R-peaks, elimination of power line interference and baseline wandering, cancellation of MECG components, removal of MECG through a deflation procedure, extraction of fECG via subspace decomposition, and derivation of the cardiotocogram or CTG (as shown in Figure 2). These steps are detailed further in the reference by (Viunytskyi and Shulgin, 2017a; Viunytskyi and Shulgin, 2017b).

Five minutes of the R-R time series were visually selected from complete CTG recordings, as illustrated in Figure 2, with each chosen complete recording exhibiting less than a 10% loss of R-peaks. This was quantified and displayed as a percentage of missing beats by the Cardiolab software, indicating beats were not detected due to poor signal quality. Additionally, we visually inspected the fECG to ensure that the selected 5-min R-R segments were continuous and free from any missing beats. The segmented R-R time series were processed with an adaptive filter proposed by Wessel et al. (2000). The filtering algorithm enhances heart rate data accuracy through three key steps. Initially, it removes implausible R-R intervals such as those of zero length, beat-to-beat intervals shorter than 200 ms—which are below the human refractory period—and significant pauses where the heart does not pump. This step ensures the basic integrity of the data. The second step employs an adaptive percent filter that adjusts for heart rate variability by smoothing the data and excluding outliers based on an adaptive mean and standard deviation, calculated from a binomial-7-filtered series. This process maintains the natural variability of the heart rate while effectively removing anomalies. Lastly, an adaptive control filter reevaluates the data to detect and correct any remaining irregularities, thereby ensuring that the final dataset accurately reflects true cardiac behavior. This systematic approach is crucial for reliable heart rate variability analysis. For implementation, a MATLAB script is freely available for download at: https://tocsy.pik-potsdam.de/ada.php.

The absence of uterine activity in the selected R-R segments was visually observed in the CTG mode of the Cardiolab software (Figure 2) and confirmed through the extraction of the electrohysterogram or uterine electromyogram using the methodology proposed by Garcia-Gonzalez et al. (2013). It is important to mention that while the women were in labor, all participants were in the latent phase, during which they experienced 0–6 cm of cervical dilation and a maximum of 1 uterine contraction every 10 min.

After the selection criteria, the total fetal continuous R-R signals were distributed as follows: the C group consisted of 22 normotensive R-R recordings, the P group included 10 recordings, and the SP group encompassed 12 recordings. The data corresponding to fetal R-R time series, their linear and nonlinear fHRV indices, and clinical data to replicate the present research results are deposited in the open-access repository of the UAM-L and can be downloaded at the following link: http://hdl.handle.net/20.500.12222/428.

The linear and nonlinear indices described in the following sections were calculated from fetal R-R signals using the open-access software PyBioS (Silva et al., 2020) and custom scripts for MATLAB (The MathWorks, Inc., Natick, MA, USA). It is worth noting that the chosen indices were selected because they are classic linear indices in HRV analysis (Malik et al., 1996; Romano et al., 2016) and symbolic nonlinear indices that were sensitive in detecting autonomic changes associated with active labor, according to a previous study conducted by our research group (Montalvo-Jaramillo et al., 2020).

### 2.3 Linear analysis of fetal heart rate variability (fHRV)

The application of different indices can provide more information, enhancing and improving the understanding of the complex dynamics involved in controlling the fetal heart rhythm. While HRV application in adults is well-established, its application in fHRV presents unique challenges due to physiological differences. Systematic reviews, notably by (Ponsiglione et al., 2021) and supported by findings in (Castro et al., 2021), have highlighted these nuances, emphasizing the need for a tailored approach to interpreting fHRV.

After preprocessing the fetal R-R time series data, we computed various time-domain indices. These included the mean value of the R-R period (referred to as mean R-R), the standard deviation of the R-R period (known as SDNN), representing the overall variability of sympathetic and vagal oscillations in the short data windows; the root mean square of the differences between successive samples (denoted as RMSSD), and the total percentage of differences between consecutive R-R intervals differing by more than 5 ms (referred to as pNN5), the RMSSD and pNN5 are indicative of parasympathetic activities, see Brändle et al., 2015; Montalvo-Jaramillo et al., 2020 for further details.

Additionally, we calculated frequency indices from the fetal R-R time series data. These included low-frequency power (LF) in the 0.05–0.2 Hz range, high-frequency power (HF) in the 0.2–1 Hz range, and the LF/HF ratio (Romano et al., 2016). An interpolation at 4 Hz was used to convert the non-equidistant beat-by-beat time series into a new time series sampled at equidistant intervals. Then, we use the Welch periodogram to calculate the power of each frequency with the Hamming window. In fHRV analysis, the interpretation of HF power and LF components requires a nuanced understanding that acknowledges the unique fetal context. HF power in fetuses primarily indicates parasympathetic activity. It can be influenced by fetal breathing movements, suggesting a potential link even without direct neural control of respiration, as seen in adults. Fetal breathing movements, detectable through frequency domain indices, induce variations in this parasympathetic activity.

Furthermore, a decrease in fetal breathing movements may signal fetal distress, highlighting the importance of HF analysis in monitoring fetal wellbeing. LF, conversely, encompasses both sympathetic and parasympathetic activities, reflecting a broader spectrum of autonomic function. This duality is crucial in assessing fetal stress and wellbeing, with increased LF associated with fetal acidemia. The LF/HF ratio, traditionally a marker of sympathovagal balance, requires careful application in fetal analysis, as it encapsulates the interplay between the sympathetic and parasympathetic nervous systems. This intricate balance is critical for understanding fetal health, as discussed in the studies by Castro et al. (2021), Cerritelli et al. (2021), and (Ponsiglione et al., 2021). To calculate these parameters, we employed a moving window approach with a fixed size of 256 heartbeats as a preprocessing step, following recommended standards (Malik et al., 1996; Grimm et al., 2003).

### 2.4 Nonlinear analysis of fHRV

The nonlinear indices calculated were symbolic dynamics, such as sigma (σ) and binary (Δ) methods, and multiscale entropy (MSE) using sample entropy (SampEn).

In symbolic dynamics, the σ and Δ methods are distinct approaches to transforming the heart rate time series into symbolic series, and both methods yield indices that are indicative of sympathetic and parasympathetic modulations (Cysarz et al., 2013). Specifically, the Δ method has been employed to measure increases or decreases in fHRV (Kurths et al., 1995; Voss et al., 2000). The series of fetal R-R values, denoted as *x*_*1*_ *, x*_*2*_ *, x*_*3*_ *, … ,x*_*N*_ were converted into a series of symbols *s*_*1*_ *,s*_*2*_ *,s*_*3*_ *, … ,s*_*N*_ with each symbol *s*_*σ,i*_ belonging to the set A based on Equation (1), where A consists of the symbols {0,1,2,3}. This conversion assigns values to one of four predefined levels relative to the average R-R interval, denoted by µ.

$${\;S}_{\sigma ,i}\left(x_{i}\right)\left\{\begin{array}{c} 0:\\ 1:\\ 2:\\ 3: \end{array}\right.\begin{array}{c} \mu \\ \left(1+a\right)\mu \\ \left(1-a\right)\mu \\ 0 \end{array}\begin{array}{c} <x_{i}\leq \left(1+a\right)\;\mu \\ <x_{i}\;<\infty \\ <x_{i}\leq \mu \\ <x_{i}\leq \left(1-a\right)\mu \end{array}$$ (1)

where *a* is a parameter setting the boundary above and below the mean by a proportion of the mean, in this research, *a* was set to 0.05, meaning the boundaries are set at 5% above and below the mean. Furthermore, a second symbolization method, the binary Δ-coding-method, was employed using “0” and “1” to represent whether the difference in R-R intervals fell below or exceeded a specified threshold:

$$S_{n}\left(x_{n}\right)\left\{\begin{array}{c} 0:\left|{RR}_{n}-{RR}_{n-1}\right|<∆ms\\ 1:\left|{RR}_{n}-{RR}_{n-1}\right|\geq ∆ms \end{array}\right.$$ (2)

where ∆ms is a predefined time interval. According to our previous study, the threshold ∆ms values were chosen as 3, 4, and 5 ms (Montalvo-Jaramillo et al., 2020). The study also classified sequences of three symbols (*k* = 3) based on the variation between consecutive symbols into categories that reflect different types of neural modulation:

- 0V% indicates no change among three consecutive symbols, suggesting solely sympathetic modulation.
- 1V% indicates a single change among three symbols, implying both sympathetic and parasympathetic modulation.
- 2LV% signifies two similar changes (either increases or decreases) among three symbols, suggesting sympathetic and parasympathetic modulation with a dominance of vagal activity.
- 2UV% denotes two dissimilar changes among three symbols, indicative of purely vagal modulation.

For binary symbolic sequences of length k = 6 (creating 64 unique binary patterns), we introduced a metric, the probability of low variability (POLVARΔ), defined as the likelihood of encountering the sequence “000000” within a symbolic string. This metric serves as an indicator of reduced fHRV. Variants of this measure, POLVAR3, POLVAR4, and POLVAR5, were analyzed, corresponding to thresholds ∆ of 3, 4, and 5 ms, respectively, to assess their effectiveness in capturing fHRV features (Montalvo-Jaramillo et al., 2020).

The quantification of regularity, irregularity, or randomness in heartbeat fluctuations can be achieved through entropy measurements, which enable the inference of complexity levels in time series such as fetal heart rate. Among these, SampEn and MSE are the most commonly used in the nonlinear analysis of fHRV (Ferrario et al., 2006; Marques et al., 2020). As a technique that decreases the bias of the approximate entropy (ApEn), the calculation of SampEn is represented by Equation (3) (Richman and Moorman, 2000):

$$SampEn\;\left(m,r,N\right)=-In\left(\frac{\phi^{m+1}\left(r\right)}{\phi^{m}\left(r\right)}\right)$$ (3)

Where ϕ represents the probability that sequences of *m* consecutive points (where *m* is the length of comparison sequences) are within a certain distance *r* (the tolerance). The probability is determined by comparing the consistency of sequences of length *m* to those of length *m*+1. In the analysis conducted by this study, *m* was set to 2, and *r* was established as 0.15 times the standard deviation (SD) of the R-R interval series, following the methodology established by Richman and Moorman (2000). On the other hand, the coarse-grained procedure was integrated, using Equation (2), which allows analysis of the fluctuations and the degree of complexity of the time series in a range of time scales, called multiscale entropy (MSE).

$$y_{j}^{\tau }=\frac{1}{\tau }\sum_{i=\left(j-1\right)\tau +1}^{j\tau }x_{i},1\leq y_{j}\leq \frac{N}{\tau }$$ (4)

In MSE, τ represents the scale factor, which is used to construct a coarse-grained time series from the original time series. For each scale factor τ, the new time series $y_{j}^{\tau }$ is created by averaging non-overlapping windows of τ consecutive data points from the original time series. The SampEn is then calculated for each of these coarse-grained time series to assess the complexity at different scales (Costa et al., 2005):

$$MSE\left(x,\tau ,m,r\right)=SampEn\left(y_{j}^{\tau },m,r\right)$$ (5)

We propose short time scales (1–5) and longer time scales (6–20); authors suggest that values of the entropy computed at the short time scales are more under parasympathetic control, whereas longer time scales should be more under sympathetic control (Silva et al., 2016).

We calculated the area under the curve using the trapezoid rule (Castiglia et al., 2023), which is known as Complexity Index (C_i_), using Equation (6):

$$C_{i}=\sum_{i=1}^{N}SE\left(i\right)$$ (6)

Where SE is SampEn values in each time scale (*i*), and *N* is the total number of time scales (Busa and van Emmerik, 2016). Although most linear and nonlinear indices have been extensively utilized within the fHRV (Gonçalves et al., 2016), assessing the C_i_ is particularly noteworthy due to its straightforward computational approach. Moreover, the distinction between short- and long-term scales has yet to be investigated in the context of preeclampsia and labor, making this approach novel and potentially relevant in the field.

Evidence that has employed MSE on heart rate variability suggests a possible decline in complexity associated with aging, conditions like atrial fibrillation, and critical illnesses such as congestive heart failure. This evidence hints at the diagnostic capabilities of MSE and may align with theories proposing a reduction in complexity as a hallmark of aging and disease development (Costa et al., 2005). The indices MSE and C_i_ were calculated using Matlab R2022b (The MathWorks, Inc, Natick, MA, USA).

### 2.5 Statistical analysis

The Shapiro-Wilk test was applied to assess the normality of the fHRV indices and the clinical characteristics of both mothers and newborns, with normality indicated by a *p*-value >0.05. To identify any significant differences between groups, both ANOVA and Kruskal Wallis tests were conducted. Given that the Kruskal Wallis tests did not reveal any significant differences across the comparisons (*p*-value >0.05), the Duncan *post hoc* test was subsequently employed following ANOVA to explore specific group differences when the ANOVA indicated a *p*-value <0.05. For protein data such as urinary creatinine, microproteins, and Mi/Cr, which were available only for women with preeclampsia, a *t*-test was performed in cases where normality was confirmed, or its non-parametric alternative was used otherwise. The R language platform (version 3.3.0) was used for the statistical analysis and data visualization.

## 3 Results

### 3.1 Clinical parameters

The maternal clinical parameters, shown in Table 1, display significant differences among the three study groups: normotensive or control (C), preeclampsia without severe features (P), and preeclampsia with severe features (SP). Post-hoc tests revealed that both SP and P groups had higher systolic blood pressure (SP: 143.17 ± 7.66 mmHg; P: 134.30 ± 4.67 mmHg) compared to the C group (116.00 ± 9.47 mmHg) with statistical significance (*p* < 0.05). Further, it was noted that both systolic and diastolic blood pressures were significantly higher in the SP group compared to the P group, emphasizing the severity of preeclampsia in the SP group. The Wilcoxon test indicated that the P group had higher levels of creatinine (123.10 ± 30.14 μmol/L) than the SP group (44.96 ± 3.29 μmol/L), and the microprotein/creatinine ratio was significantly greater in the SP group (0.82 ± 0.10) compared to the P group (0.30 ± 0.02) with *p*-values <0.05. Platelet counts were notably lower in the SP group (168.00 ± 31.82 × 10^3^/μL) than in the C group (219.91 ± 59.50 × 10^3^/μL) and the P group (230.80 ± 40.04 × 10^3^/μL) with statistical significance (*p* < 0.05).

**TABLE 1:** Mean (±SD) of the maternal clinical parameters of participants: normotensive (C), preeclampsia without severe features (P), and preeclampsia with severe features (SP).

*Clinical parameters*	*C (n = 22)*	*P (n = 10)*	*SP (n = 12)*
Age [years]	26.57 ± 6.56	22.40 ± 4.57	27.50 ± 8.43
Gestational age [weeks]^a^ ^,^ ^b^	38.8 ± 1.3	37.6 ± 1.4	36.9 ± 1.2
BMI [kg/m^2^]	29.20 ± 5.04	30.20 ± 5.84	28.37 ± 4.31
Systolic blood pressure [mmHg]^a^ ^,^ ^b^ ^,^ ^c^	116.00 ± 9.47	134.30 ± 4.67	143.17 ± 7.66
Diastolic blood pressure [mmHg]^a^ ^,^ ^b^ ^,^ ^c^	71.68 ± 8.96	83.80 ± 7.81	92.83 ± 4.86
Leukocytes [10^3^]	9.25 ± 3.80	9.92 ± 2.19	8.77 ± 1.74
Erythrocytes [10^6^]	4.38 ± 0.31	4.34 ± 0.57	4.42 ± 0.50
Hemoglobin [g/dL]	13.25 ± 1.44	12.28 ± 1.43	13.02 ± 1.64
Hematocrit [%]	39.49 ± 4.17	37.01 ± 4.08	38.84 ± 4.28
Platelets [10^3^/μL]^a^ ^,^ ^c^	219.91 ± 59.50	230.80 ± 40.04	168.00 ± 31.82
Urinary creatinine [µmol/L] ^c^	-	123.10 ± 30.14	44.96 ± 3.29
Microproteins [mg/dL] (Mi)	-	36.89 ± 11.05	36.61 ± 4.13
Mi/Cr ratio^c^	-	0.30 ± 0.02	0.82 ± 0.10

^a^ *p* < 0.05 between C vs. SP.

^b^ *p* < 0.05 between C vs. P.

^c^ *p* < 0.05 between SP vs. P.

Table 2 illustrates the neonatal clinical parameters. Notably, the abdominal perimeter was significantly larger in the SP group (29.29 ± 2.24 cm) compared to the P group (31.10 ± 1.66 cm) and the C group (30.80 ± 1.51 cm) with a *p*-value <0.05.

**TABLE 2:** Mean (±SD) of the clinical parameters of newborns: normotensive (C), preeclampsia without severe features (P) and preeclampsia with severe features (SP).

*Clinical parameters*	*C*	*P*	*SP*
Newborn weight [g]	3068.79 ± 346.40	2798.10 ± 451.10	2932.90 ± 386.19
Size [cm]	47.86 ± 2.44	48.66 ± 1.25	48.33 ± 2.62
APGAR 1 min [points]	7.92 ± 0.27	7.92 ± 0.32	8.00 ± 0.00
APGAR 5 min [points]	9.00 ± 0.00	9.00 ± 0.00	9.00 ± 0.00
Head circumference [cm]	34.60 ± 1.27	34.00 ± 1.25	33.71 ± 1.45
Thorax circumference [cm]	33.20 ± 1.58	33.00 ± 2.11	32.46 ± 2.42
Abdominal perimeter [cm]^a^ ^b^	30.80 ± 1.51	31.10 ± 1.66	29.29 ± 2.24

^a^ *p* < 0.05 between C vs. SP.

^b^ *p* < 0.05 between SP vs. P.

### 3.2 Linear indices

Table 3 summarizes the linear indices of short-term fetal heart rate variability (fHRV). Significant differences were found using ANOVA, with the P group showing higher SDNN values (14.16 ± 5.59 ms) compared to the C group (9.39 ± 3.95 ms) with a *p*-value <0.05. Similarly, for LF, significant differences were observed; the P group had higher LF values (34.9 ± 16.4 ms^2^) compared to the C group (19.25 ± 14.34 ms^2^) and the SP group (22.88 ± 14.17 ms^2^), with a *p*-value <0.05 for comparisons between both C vs. P and SP vs. P

**TABLE 3:** Mean (±SD) of the linear indices of short-term fetal heart rate variability (fHRV).

Linear indexes	C	P	SP
SDNN [ms]^a^	9.39 ± 3.95	14.16 ± 5.89	112.97 ± 5.95
RMSSD [ms]	3.75 ± 1.30	4.38 ± 1.47	3.77 ± 0.97
R-R mean [ms]	437.48 ± 22.24	426.58 ± 29.16	423.78 ± 25.88
pNN5 (%)	11.59 ± 8.85	14.32 ± 8.95	12.05 ± 6.23
LF [ms^2^] ^a^	19.25 ± 14.34	34.90 ± 16.44	22.88 ± 14.17
HF [ms^2^]	5.21 ± 4.04	7.99 ± 4.43	5.61 ± 3.37
LF/HF [units]	3.83 ± 2.03	4.98 ± 2.61	4.78 ± 2.77

^a^ *p* < 0.05 between C vs. P.

### 3.3 Nonlinear indices

**FIGURE 3:**
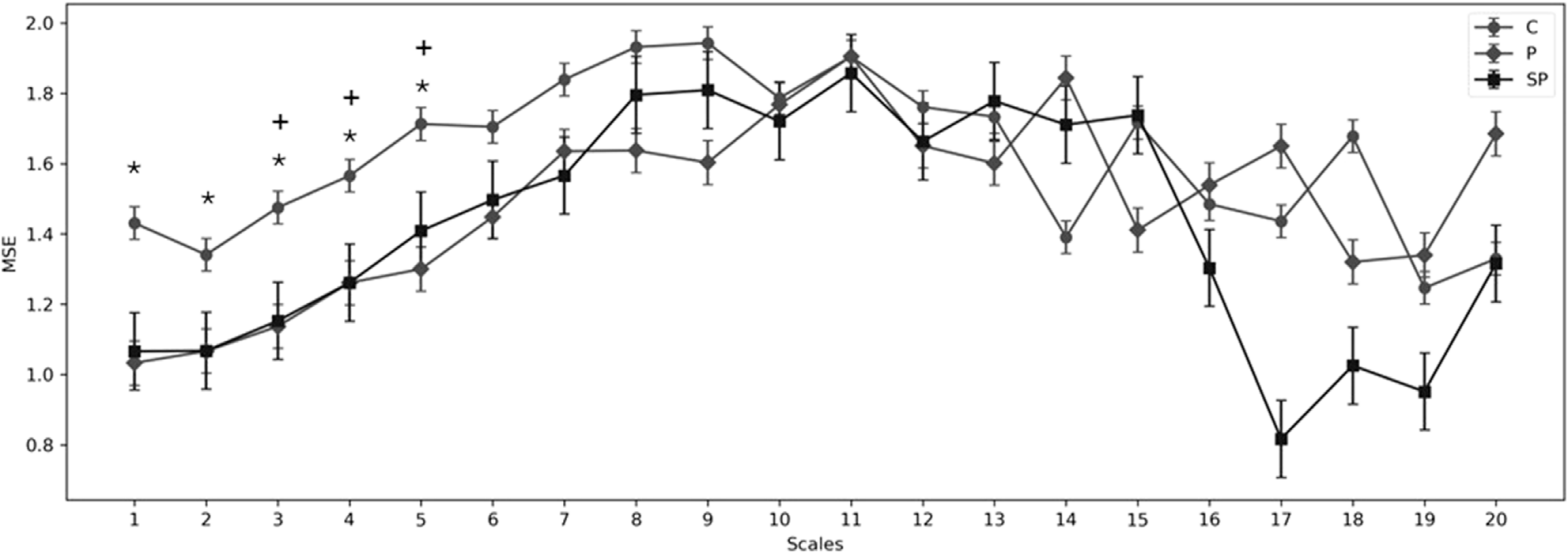
Multiscale entropy (MSE) for scales 1–20 for the C (normotensive or control), preeclampsia with moderate features (P), and preeclampsia with severe features (SP). Showing significant differences (*p* < 0.05) in the groups: *p < 0.05 between C vs. SP; +*p* < 0.05 between C vs. P.

Table 4 shows significant differences in nonlinear indices such as MSE and C_i_ when comparing the C and PE groups (P and SP). For the short scales (1–5) of the C_i_, the C group values were greater than those in both P and SP groups, while for the larger scales (6–20) of the C_i_ did not show significant differences. Consistently, Figure 3 shows significant differences in the shorter scales of MSE, with the pre-eclamptic groups exhibiting lower entropy values compared to the Control group.

**TABLE 4:** Mean (±SD) of the nonlinear indices of short-term fetal heart rate variability (fHRV).

Nonlinear indexes	C	P	SP
0V%	75.99 ± 6.01	76.61 ± 5.72	78.59 ± 7.80
1V%	18.17 ± 4.66	18.77 ± 4.44	16.33 ± 5.69
2UV%	5.76 ± 1.82	4.51 ± 1.82	5.01 ± 2.61
2LV%	0.07 ± 0.13	0.11 ± 0.15	0.07 ± 0.12
POLVAR3	0.30 ± 0.14	0.32 ± 0.12	0.33 ± 0.15
POLVAR4	0.47 ± 0.17	0.43 ± 0.16	0.48 ± 0.17
POLVAR5	0.60 ± 0.17	0.55 ± 0.17	0.60 ± 0.17
SampEn	1.43 ± 0.51	1.03 ± 0.39	1.07 ± 0.50
C_i_ (1–5)^a^ ^b^	5.95 ± 1.51	4.63 ± 1.62	4.72 ± 1.59
C_i_ (6–20)	23.37 ± 4.21	22.47 ± 5.06	21.14 ± 4.42

^a^ *p* < 0.05 between C vs. SP.

^b^ *p* < 0.05 between C vs. P.

## 4 Discussion

Our findings reveal significant differences in nonlinear indices such as MSE and C_i_ between the normotensive and preeclampsia groups (P and SP). These differences are particularly notable in the MSE values across various short-term scales, showing lower complexity in the fetal heart rate time series for the PE groups than the control group. This reduction in complexity (confirmed by reduced short-term C_i_) aligns with the results of Costa et al. (2014), who found that the complexity of fetal heart rate signals, as measured by MSE, was significantly lower in acidemic fetuses than in non-acidemic ones. Costa et al.'s study supports that decreased nonlinear complexity is a dynamical signature of impaired physiological control systems. Our observations of reduced complexity in the PE groups potentially indicate a similar physiological disruption, hinting at fetal acidemia’s presence, especially in the context of labor. Although our results suggest a potential association between the PE group and fetal acidemia, it is crucial to note that further confirmation is required. Clinical studies are needed to establish this relationship definitively and evaluate the practical utility of entropy analysis in early fetal acidemia detection, as Spilka et al. (2012). Our findings underscore future research’s importance in enhancing fetal monitoring and improving clinical care.

A significant difference in the MSE of short scales (1–5) was found in the P and SP groups compared to the C group. For short scales, respiratory modulation of heart rate can be detected since greater amplitude has been seen in healthy subjects compared to some cardiovascular diseases (Costa et al., 2005), while in healthy fetuses, Schneider et al. (2018) have reported respiratory movements related to the high complexity in the time series, whose respiratory movements have been associated with the coupling of respiratory sinus arrhythmia (RSA) (Schneider et al., 2018; Zizzo et al., 2022). This could indicate that P and SP could have lower coupling RSA compared to the normotensive group (Lakhno, 2016; Lakhno, 2017). The RSA in fetuses suggests early autonomic system regulation, marked by RSA-like heart rate fluctuations during fetal respiratory movements (FRMs), indicating vagal modulation. Studies by Zizzo et al. (2020) and Zizzo et al. (2022) emphasize FRMs’ role in modulating fHRV, with observed increases in RMSSD and HF-power, highlighting RSA-like activity despite no actual respiration.

In the temporal linear analysis of fHRV, significant differences were observed in the SDNN index between Groups C and P, with higher values in the P group. It has been reported that in healthy conditions, the increase in the SDNN index as the weeks of gestation weeks progress, indicating an increase in the control of autonomic modulation of the fetus, is also associated with the overall variability of the neurovegetative system. An increase in fetal sympathetic activation is also linked to enhanced parasympathetic modulation capacity, particularly during the transition from the second to the third trimester (Schneider et al., 2018). Conversely, reduced values in the SDNN index have been related to potential parasympathetic dysfunction in pregnant women diagnosed with severe preeclampsia, leading to increased sympathetic tone in fetuses and the development of fetal distress (Lakhno, 2017). The literature suggests that a well-developed fetus in normotensive women can adapt to changes induced by labor, such as initiating spontaneous breathing (Morton and Brodsky, 2016). However, in challenging birth scenarios, the fetus may experience respiratory issues and difficulties in autonomic regulation post-birth (Hillman et al., 2012). Our results indicate that group P, when faced with labor stress, may exhibit a higher overall fluctuation range than fetuses in the C group. The behavior of the SP group appears similar to that of group C, suggesting the need to explore additional indices to better understand the SP group’s behavior. Lakhno, 2017 reported that the severity of PE in the third trimester is inversely related to the SDNN index compared to the normotensive group. During severe cases at birth, an increase in fHRV is observed in the fetus, escalating the risk of complications in the newborn (Tarvonen et al., 2022).

The findings from our study, particularly the observed changes in LF among the preeclampsia groups, suggest a nuanced adaptive response to the labor process. The elevated LF values could imply an increase in sympathetic activity, alongside a possible rise in parasympathetic activity, as part of the fetus’s adaptation to the stressors associated with preeclampsia during labor. This increase in sympathetic activity aligns with previous findings by Montalvo-Jaramillo et al. (2020), who reported similar trends in healthy fetuses during labor. Additionally, the potential increase in parasympathetic activity could be related to mechanisms previously described by Frasch et al. (2016), which are associated with the activation of the cholinergic anti-inflammatory pathway from a neuroinflammatory perspective. These combined autonomic responses highlight the complex interplay between sympathetic and parasympathetic systems in fetuses undergoing labor in the context of preeclampsia, underscoring the adaptive capacities of the fetal autonomic nervous system in response to intrauterine stress conditions.

Our clinical findings reveal differences in the abdominal perimeter (AP) among neonates from different groups. Specifically, the AP was significantly larger in the P group compared to the SP group. This variation in AP is a critical indicator, especially considering the broader context of neonatal health and development. It is interesting to note that low birth weight is a common observation in healthy neonates at higher altitudes, as (Bailey et al., 2019) reported. In a related context, our study was conducted at the same hospital in Toluca where Nava Díaz et al. (2012) also carried out their research, indicating neonatal weights similar to those we found in our control group.

Consequently, altitude could significantly influence the analysis of AP in neonates. The AP is a valuable parameter for assessing the nutritional status of a neonate before birth. Under normotensive conditions, an AP below the 10th percentile (34.11 cm) often suggests malnutrition, as detailed by Padilla-Amigo et al. (2022). Furthermore, in neonates affected by preeclampsia, a smaller AP, specifically below the 3rd percentile (33.14 cm), has been linked to early intrauterine growth restriction (Gordijn et al., 2016). Our results showed that the percentile 3^rd^ of the AP measurements in neonates from the PE groups were 31.10 cm in the P group and 29.29 cm in the SP group. Both measurements are below the 3^rd^ percentile, indicating a potential relation to early intrauterine growth restriction. This observation aligns with the findings of Llanos Buelvas et al. (2011) and highlights the impact of preeclampsia on fetal development. These findings underscore the need for careful monitoring and management of pregnancies complicated by preeclampsia to mitigate risks associated with intrauterine growth restriction.

In the clinical results concerning the systolic and diastolic blood pressure of the pregnant women, the values were significantly higher in the P and SP groups compared to the C group. During labor, maternal blood pressure in normotensive pregnant women is expected to increase, with SBP reaching up to 136 mmHg and DBP up to 82 mmHg (Cohen et al., 2015). Compared to our results in the C group, these expected values fall within the 5th and 10th percentile, as reported by Cohen et al. In pregnant women diagnosed with preeclampsia, an increase in blood pressure up to 159 mmHg for SBP and 99 mmHg for DBP is anticipated, although only 9% of the population reported by Cohen et al. (2015) had these parameters. Furthermore, similar SBP and DBP results have been observed in pregnant women receiving hypertensive treatment (Furukawa et al., 2006), suggesting that high blood pressure may not be exclusively associated with preeclampsia. In analyzing other parameters for the diagnosis of preeclampsia, we noted a lower platelet count in the PE groups compared to the C group. This decrease in platelet count is associated with endothelial damage and vascular dysfunction in spiral arteries (Pacheco-Romero, 2017).

Regarding creatinine levels in urine, our results indicated higher values in the P group compared to the SP group, a finding typically associated with renal failure (Traferri et al., 2021). Thus, the P group showed a higher probability of renal failure than the SP group. However, these findings contrast with previous reports suggesting higher creatinine levels in the SP groups compared to the P groups (Furukawa et al., 2006). We also observed a higher protein/creatinine ratio in the SP group compared to the P group. Despite being an indicator of preeclampsia severity, with preeclampsia being greater than 0.3 mg/dL meeting severity criteria (Walker et al., 2024), the protein/creatinine ratio has also been related to this disease (Traferri et al., 2021). This highlights the complexity of managing and diagnosing preeclampsia, with various factors contributing to its severity and manifestation.

From a clinical perspective, these insights offer a speculative yet potential application: the development of targeted intervention strategies for neonates born under these high-risk conditions. For instance, recognizing the specific autonomic behavior of fetuses not diagnosed with PE until labor could inform the creation of tailored monitoring and treatment protocols immediately post-birth, aimed at mitigating the adverse effects of this altered autonomic state. Such interventions could be critical for improving the outcomes of neonates subjected to the stress of PE during labor, particularly in cases where PE is undiagnosed until the onset of labor and no antihypertensive treatment has been administered during pregnancy.

## 5 Limitations

In revising the limitations section of our study, we acknowledge the absence of a direct evaluation of noise contribution within our study’s framework. Nonetheless, meticulous protocols were carefully followed to mitigate the impact of noise and unstable conditions during the recording phase. These included comprehensive skin preparation procedures and the use of the BabyCard device, which boasts technical requirements like an Input Impedance >50 MOhm and a CMRR >120dB at 50 Hz. These specifications were instrumental in ensuring the quality of fetal ECG extraction, even in challenging conditions such as intrapartum, by facilitating algorithms designed to maintain signal integrity (Viunytskyi and Shulgin, 2017a). This approach allows for the acceptance of signals with acceptable quality (notably, with less than a 10% loss of fetal heartbeat) while minimizing the effects of high-amplitude noise and artifacts. Although stationarity was not directly assessed, the PyBios software implements a detrending process aimed at evaluating the nonstationary components of the R-R signal, thereby removing the slow nonstationary trends from the fHRV time series before analysis through a constant detrending method according to PyBioS (Silva et al., 2020). This step was critical in power spectral estimation, ensuring the analysis was not significantly affected by nonstationary trends.

An additional limitation of our study stems from the lack of medical history prior to the onset of labor for our participants. Specifically, the absence of data regarding the diagnosis timing and duration of elevated blood pressure and proteinuria in the participants could potentially influence our results, particularly in the context of fHRV analysis. Without these historical health metrics, it is challenging to fully understand the extent to which pre-labor conditions of preeclampsia might have affected our findings. This limitation highlights the need for further research incorporating comprehensive pre-labor medical histories to elucidate the relationship between the duration and severity of preeclampsia symptoms before labor and their impact on fHRV outcomes.

Following worldwide guidelines that recommend inducing labor before the 37th week of pregnancy for women with preeclampsia (Vidaeff et al., 2019), the majority of participants with preeclampsia were medically advised to have a C-section. This medical guidance led to significant differences in the mean gestational ages across the study groups, as shown in Table 1, potentially impacting the outcomes of our fHRV analysis. However, it is important to note that all subjects in the study were in their third trimester (>36 weeks of gestation), suggesting that the primary alterations observed might be primarily linked to the condition of preeclampsia.

Furthermore, we acknowledge the limited number of subjects. Our decision on the participant count was influenced by prior research, specifically the study by Pichardo-Carmona et al. (2023), which successfully identified autonomic differences in preeclamptic women during labor, and the work by Montalvo-Jaramillo et al. (2020) that analysed fHRV in active labor. These previous studies supported the adequacy of our sample size for the exploratory objectives at hand. It is crucial to emphasize the exploratory nature of our research, aimed at generating initial insights into a relatively underexplored area. Moving forward, we plan to expand our sample size in future studies to build on these preliminary findings and enhance the robustness of our conclusions.

## 6 Conclusion

Our study highlights the changes in fetal heart rate complexity and fetal autonomic cardiac activity in women with preeclampsia (PE) during labor, uniquely focusing on those not receiving treatment during pregnancy. We observed significant differences in the complexity of the beat-to-beat fetal heart rate time series between the control (C) and PE groups using nonlinear indices (MSE, C_i_). Notably, the lower complexity observed in the PE groups might be associated with reduced adaptability to adverse conditions. Fetuses in the group with moderate features of preeclampsia exhibited possible autonomic modifications compared to the control or normotensive group. These modifications are evidenced by alterations in low frequency (LF) indices, suggesting a complex autonomic response, likely reflecting both an increase in sympathetic activity and parasympathetic involvement during PE. This nuanced autonomic adjustment could be an adaptive mechanism to counterbalance the challenging conditions posed by preeclampsia and labor. Given these insights, and considering our study is among the few involving women with PE who have not received treatment during pregnancy and are at the onset of labor, future research should delve deeper into the varying stages of preeclampsia during pregnancy. Additionally, exploring different types of entropy for a more comprehensive understanding of signal complexity in this context is recommended.

## Data Availability

The original contributions presented in the study are publicly available. This data can be found here: http://hdl.handle.net/20.500.12222/428 or https://xogi.ler.uam.mx/items/c38ba507-24de-4f9b-9079-92a7745b7b8b.
